# Coexistence of Adult-Onset Still's Disease and Graves' Disease: Coincidence or Continuum?

**DOI:** 10.7759/cureus.100076

**Published:** 2025-12-25

**Authors:** Inass Chaari, Ayoub Idrissi, Lahoussaine Abainou, Hamza El Jadi, Azzelarab Meftah, Hicham Baizri

**Affiliations:** 1 Endocrinology and Diabetology, Avicenna Military Hospital, Marrakech, MAR; 2 Endocrinology, Diabetes and Metabolism, Avicenna Military Hospital, Marrakech, MAR; 3 Endocrinology, Oued Eddahab Military Hospital, Agadir, MAR; 4 Biosciences and Health Laboratory, Faculty of Medicine and Pharmacy, Cadi Ayyad University, Marrakech, MAR

**Keywords:** adult-onset still’s disease, glycosylated ferritin, graves-basedow, rare coexistence, still

## Abstract

Adult-onset Still’s disease (AOSD) is a rare systemic autoimmune disorder of unclear etiology, typically characterized by prolonged fever, arthralgia, transient rash, and leukocytosis. Graves’ disease (GD) is a common autoimmune cause of hyperthyroidism in young adults. Although associations between autoimmune conditions have been reported, the coexistence of AOSD and GD remains uncommon.

We describe the case of a 22-year-old patient presenting with clinical and biochemical hyperthyroidism, confirmed by thyroid scintigraphy. The clinical picture was further complicated by persistent inflammatory syndrome, marked hyperferritinemia, and hepatosplenomegaly, consistent with AOSD as defined by Yamaguchi’s criteria. The disease course was characterized by recurrent inflammatory flares of both conditions and the development of carbimazole-induced hepatocellular injury, necessitating discontinuation of the drug and subsequent radioiodine ablation. The patient was also treated with corticosteroid therapy, leading to favorable clinical and laboratory improvement.

Several reports have documented the association between AOSD and GD, suggesting a potential shared pathophysiological basis. Both conditions exhibit genetic susceptibility linked to HLA-DRB1 alleles and share immune dysregulation driven by excessive cytokine production, particularly interleukin-18, tumor necrosis factor-α, and interferon-γ. This molecular overlap may account for their coexistence in some patients, regardless of sex.

Systematic screening for thyroid dysfunction is advisable in patients with AOSD, irrespective of gender, in order to better identify this underrecognized association.

## Introduction

Adult-onset Still’s disease (AOSD) is a rare systemic autoimmune disorder of uncertain etiology. Graves’ disease (GD) is an autoimmune thyroid condition and represents the leading cause of hyperthyroidism in young adults.

An association between autoimmune thyroid disorders and rheumatoid arthritis has previously been documented [1]. Since AOSD is regarded as a clinical variant of rheumatoid arthritis [2], it may also coexist with autoimmune hyperthyroidism in some patients, as well as with myasthenia gravis, systemic lupus erythematosus [3], and Sjögren’s syndrome [4].

We report a case of AOSD occurring concomitantly with GD, suggesting that auto-inflammatory processes may act as a potential trigger for recurrent thyroid dysfunction. Beyond the rarity of this coexistence, our observation underlines the importance of systematic thyroid function screening in patients with AOSD [5,6].

## Case presentation

We report the case of a 22-year-old male with no significant past medical history who presented with a three-month history of polyarthralgia associated with unquantified weight loss and recurrent fever. There was no cutaneous rash or odynophagia. Cervical examination revealed a diffusely enlarged thyroid gland of elastic consistency, without palpable nodules or cervical lymphadenopathy. Notably, clinical assessment did not demonstrate exophthalmos.

**Figure 1:**
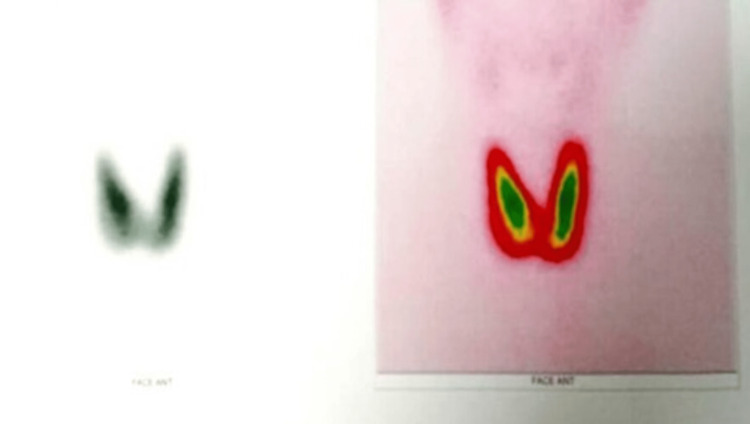
Acquisition of a static anterior image centered on the cervical region, 20 minutes after injection of 5 mCi of 99 mT Thyroid scintigraphy demonstrating an enlarged gland with diffuse, homogeneous, and markedly increased tracer uptake, consistent with Graves’ disease.

Biological investigations supported the diagnosis of GD, with suppressed thyroid-stimulating hormone (TSH) less than 0.008 mU/L (reference: 0.25-5), markedly elevated free T4 at 309 pmol/L (≈15× upper normal limit; reference: 10.6-19.4), and negative anti-TSH receptor antibodies at 1.10 UI/L (reference: <1.17). Thyroid scintigraphy revealed diffuse, homogeneous, and markedly increased uptake throughout an enlarged thyroid gland (Figure 1). Cervical ultrasonography confirmed a diffuse, hypervascular goiter without nodular lesions, consistent with GD.

The patient was started on carbimazole at 40 mg/day. After six weeks of therapy, he developed signs of drug-induced hepatotoxicity, with cytolysis evidenced by aspartate aminotransferase (AST) at 208.3 U/L (≈4.6× normal) and alanine aminotransferase (ALT) at 254.8 U/L (≈5.6× normal), as well as cholestasis with alkaline phosphatase (ALP) at 628.1 U/L (≈5× normal) and gamma-glutamyl transferase (GGT) at 667.2 U/L (≈14.8× normal). In light of these abnormalities, carbimazole was discontinued. Viral hepatitis serologies were negative (Table 1).

**Table 1:** Evolution of laboratory findings and treatment timeline Sequential laboratory results showing thyroid, hepatic, and inflammatory parameters from February to June 2024. Liver enzyme elevation occurred after carbimazole therapy, which normalized following drug withdrawal and corticosteroid treatment. AST, Aspartate aminotransferase; ALT, Alanine aminotransferase; TSH, Thyroid-stimulating hormone; CRP, C-reactive protein

Date	Leukocytes (NR: 4000-11000 /mm³)	Free T4 (pmol/L) (NR: 12-22)	TSH (mU/L) (NR: 0.25-5)	AST (U/L) (NR: <50)	ALT (U/L) (NR: <65)	CRP (mg/L) (NR: <5)	Treatment received
February 17, 2024	10650	309	0.008	29	29.8	Not available	Carbimazole 40 mg/day
March 6, 2024	12400	40.77	Not available	76	45	66.8	Treatment discontinued
April 9, 2024	14300	7.32	Not available	208.39	254.84	116.8	Prednisolone 60 mg/day
May 2, 2024	8600	14.8	Not available	67.5	120.4	14	
June 2, 2024	7500	19.91	3.68	21	29	Not available	

Given the presence of persistent fever, laboratory testing revealed markedly elevated C-reactive protein at 116.8 mg/L, leukocytosis of 14,300/mm³, increased erythrocyte sedimentation rate, and elevated lactate dehydrogenase at 640 U/L. An etiological workup was undertaken: urine culture was sterile, blood cultures obtained during febrile spikes were negative, the Quantiferon test excluded latent tuberculosis infection, and serum protein electrophoresis confirmed an inflammatory profile.

In the context of suspected AOSD, alternative diagnoses were systematically ruled out. Rheumatoid factor, anti-cyclic citrullinated peptide antibodies, and antinuclear/cytoplasmic antibodies all tested negative.

**Figure 2:**
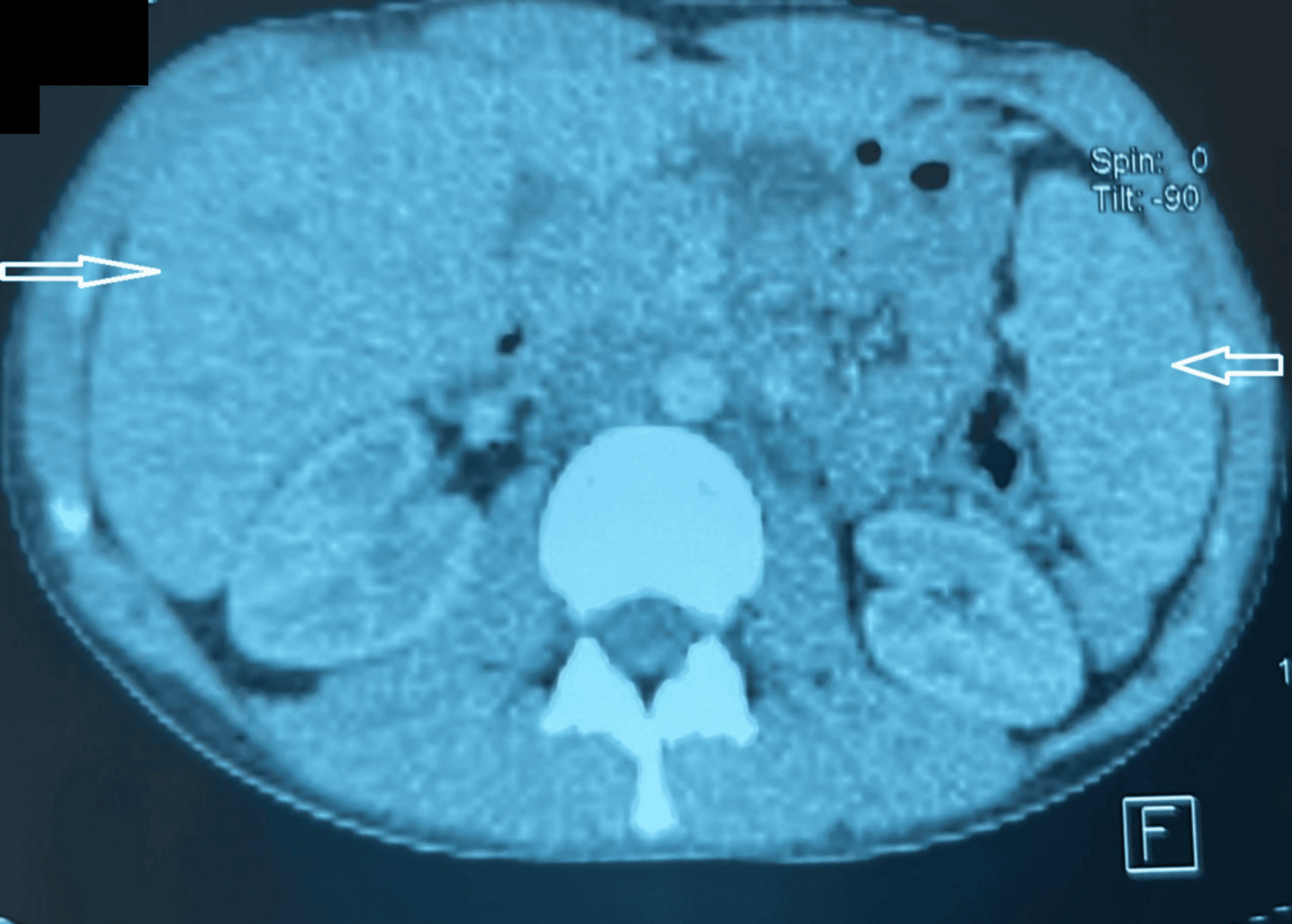
Thoracoabdominal CT showing homogeneous hepatosplenomegaly Axial contrast-enhanced abdominal CT demonstrating homogeneous enlargement of the liver and spleen (arrows). CT, Computed tomography

**Figure 3:**
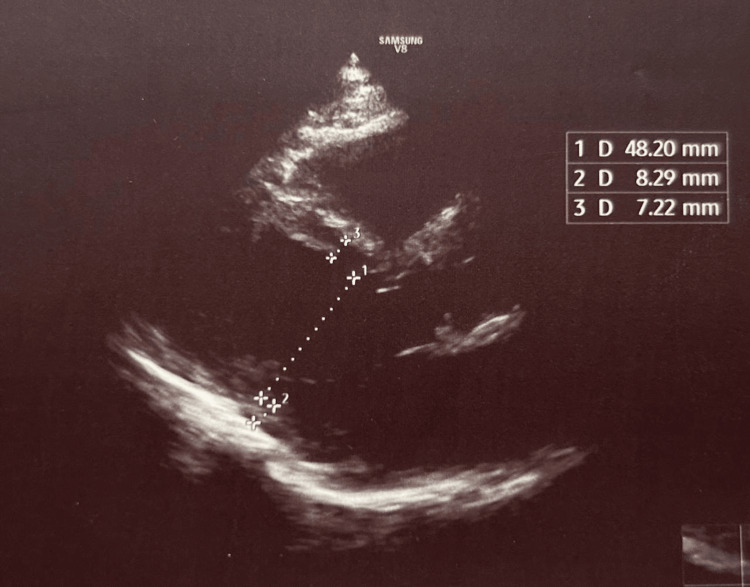
Transthoracic echocardiography showing normal cardiac morphology and function Parasternal long-axis transthoracic echocardiographic view showing normal cardiac chamber dimensions and wall thickness. Linear measurements are indicated.

Imaging investigations showed homogeneous hepatosplenomegaly on thoraco-abdominal computed tomography (CT) scan (Figure 2), while transthoracic echocardiography was unremarkable (Figure 3).

In the absence of evidence for infectious or malignant disease, and given the constellation of clinical and biological findings, the diagnosis of AOSD was considered according to the Yamaguchi criteria [7]. Supporting this, serum ferritin was >1200 ng/mL, with glycosylated ferritin reduced to 13%.

The patient was started on corticosteroid therapy with prednisolone 60 mg/day, resulting in a favorable initial clinical and biological response.

The subsequent evolution of the patient was marked by alternating flares and remissions of both conditions. Due to recurrent hepatocellular injury, definitive management of GD with radioiodine therapy was proposed. The patient subsequently received 20 mCi of radioactive iodine.

## Discussion

The coexistence of AOSD and GD has been previously documented. In Japan, Torigoe et al. (2011) described the case of a 50-year-old woman with AOSD and recurrent GD, raising the possibility of shared pathophysiological mechanisms [6]. In their report, the authors also referred to five additional cases of concurrent presentation (Table 2).

**Table 2:** Reported cases of concomitant Graves’ disease and adult-onset Still’s disease Summary of published cases describing the coexistence of Graves’ disease and adult-onset Still’s disease, including country of origin, year of report, patient demographics, and response to corticosteroid therapy.

Authors (references)	Country	Year	Patient age	Patient sex	Response to treatment (corticosteroids)
Hu et al. [5]	China	2014	43 years	Female	Good response
Torigoe et al. [6]	Japan	2011	50 years	Female	Good response
		1995	47 years	Female	Good response
		1996	52 years	Female	Good response
		1996	26 years	Female	Good response
		2001	30 years	Female	Good response
Chen et al. [8]	Taiwan	2010	37 years	Female	Good response

In 2014, Hu et al. reported another case of AOSD associated with thyroid dysfunction in a 43-year-old woman with preexisting GD, whose course was complicated by active AOSD [5]. The same study cited a similar observation previously published by Chen et al. [8].

These findings revived the debate on whether a pathophysiological link exists between the two diseases and highlighted the importance of reciprocal screening.

Although the pathogenesis of both AOSD and GD is complex and multifactorial, several overlapping features have been identified. From a genetic perspective, both conditions are associated with certain HLA-DRB1 alleles. Immunologically, a predominance of T-helper 1 (Th1)-mediated immune response, characterized by elevated levels of tumor necrosis factor-α (TNF-α), soluble TNF receptor 1, interleukin (IL)-6, and IL-18, plays a central role in AOSD pathogenesis.

IL-18, in particular, has been recognized as a key mediator within the inflammatory cascade, capable of inducing other cytokines such as IL-1β, which alters the tight junction integrity of human thyrocytes, along with IL-8, TNF-α, and interferon-γ (IFN-γ) [9].

Moreover, high serum levels of C-X-C motif chemokine ligand 10 (CXCL10), an IFN-γ-inducible chemokine, have been reported in both AOSD and GD patients, compared to healthy controls and other autoimmune disorders [10,11].

## Conclusions

This case highlights the rare coexistence of AOSD and GD in a young patient and adds to the limited number of similar reports in the literature. The diagnosis was supported by compatible clinical, biological, and imaging findings, and by the exclusion of infectious, malignant, and other autoimmune causes. Although a shared immunoinflammatory background between these two conditions has been suggested, the exact relationship remains unclear. Further studies are needed to determine whether this association is coincidental or reflects common pathogenic pathways, and to clarify its potential diagnostic and therapeutic implications.
